# Perceptions of family physicians in Istanbul about e-cigarettes as smoking cessation aids: a qualitative study

**DOI:** 10.1186/s13722-024-00532-z

**Published:** 2024-12-30

**Authors:** Melis Selamoglu, Ayse Emel Onal, Bircan Erbas, Selma Karabey, Meryem Merve Oren, Mahmut Talha Ucar, Chris Barton

**Affiliations:** 1 Department of General Practice, School of Public Health and Preventive Medicine, Monash University, 553 St Kilda Rd, Level 5, Prahran, Melbourne, VIC 3181 Australia; 2 Department of Public Health, Faculty of Medicine, Istanbul University, Istanbul, Türkiye; 3 Department of Public Health, School of Psychology and Public Health, La Trobe University, Melbourne, Australia; 4 Department of Public Health, Hamidiye Faculty of Medicine, University of Health Sciences, Istanbul, Türkiye

**Keywords:** E-cigarette, Smoking cessation, Harm reduction, Family physician, General practice, Primary care, Public health, Türkiye

## Abstract

**Background:**

Family physicians (FPs) are the first point of contact for people who smoke who are seeking to quit smoking in Türkiye. We aimed to explore Turkish FPs knowledge, attitudes and perceptions of e-cigarettes as smoking cessation aids.

**Methods:**

Eleven in-depth semi-structured interviews were conducted with FPs in Istanbul, Türkiye. Participants were recruited through purposeful sampling from respondents to a previous survey study completed with 243 participants in 2022. The survey explored the knowledge, attitudes and recommendations of FPs in Istanbul about e-cigarettes as smoking cessation aids. Participants indicated availability for a follow up qualitative interview. FPs were interviewed and audio files were transcribed verbatim. QSR NVivo was used to aid coding, thematic analysis and retrieval of quotes.

**Results:**

FPs expressed they had little knowledge about e-cigarettes and this impacted their confidence to discuss these with their patients. They held a range of views on the harms of e-cigarettes and the potential for a gateway effect and dual use with tobacco cigarettes. FPs stated they would not recommend e-cigarettes as smoking cessation aids and half were not keen on a prescription model for e-cigarettes. FPs did not feel comfortable or have the confidence to answer patient questions about e-cigarettes if asked. They were keen to learn more about e-cigarettes and receive training and education to be able to better inform their patients about e-cigarettes as smoking cessation aids.

**Conclusion:**

FPs in Türkiye require more understanding of e-cigarettes if they are to answer patient questions about using e-cigarettes to support smoking cessation. They perceived to be lack of evidence and research on the harms of vaping and as such were not currently willing to recommend them to patients. FPs desired more information and resources from trusted sources to support them to learn about e-cigarettes in order to discuss them with patients. Providing evidence-based information and upskilling FPs on e-cigarettes may increase their knowledge and confidence to have discussions about e-cigarettes for smoking cessation.

**Supplementary Information:**

The online version contains supplementary material available at 10.1186/s13722-024-00532-z.

## Background

The global tobacco epidemic is responsible for more than 8 million deaths every year from smoking related illnesses and 7 million deaths have a direct link to tobacco use [1]. In Türkiye alone, approximately 100,000 citizens die from tobacco related illnesses each year and is estimated to reach 240,000 by 2030 [2, 3].

Despite Türkiye being one of the first nations in the world to adopt the World Health Organization (WHO) Framework Convention on Tobacco Control (FCTC) in 2004 and is listed as one of the ‘*best practiced countries’* in the world [4], the current prevalence of cigarette smoking in Türkiye is 29.1% [3, 5] compared to other European nations such as the UK (11.4%) and Sweden (9%) [5]. Türkiye introduced significant tobacco control measures since adopting the FCTC guidelines and in January 2020 implemented the tobacco plain packaging policy [6].

Electronic cigarettes (e-cigarettes) are devices that heat nicotine liquids (e-liquid) and other substances that produce an aerosol which is inhaled (vaped) by the user [7, 8]. Currently in Türkiye, the sale (both in tobacco stores and online), marketing, distribution and use of e-cigarettes indoors as well as any e-cigarette parts or accessories (solutions) are prohibited [9]. Despite the ban on sales of e-cigarettes, vaping is legal in Türkiye and individuals are able to obtain e-cigarettes and e-liquids via online sellers or by importing them from abroad for personal use [10]. According to Cakir (2023), the prevalence of daily and occasional e-cigarette use is 0.4% among individuals aged 15 years and over [11].

Family physicians (FPs) have the responsibility of assessing every patient for tobacco dependence and is a standard procedure in everyday clinical settings [12]. They can encourage, motivate and support patients to quit smoking by providing up to date relevant information, referrals and treatment management [7]. In Istanbul 4,500 FPs work across 1,200 family medicine clinics [13] and take care of a population recorded to be 16 million people [14]. In Türkiye, FPs are able to refer patients who smoke to smoking cessation clinics in order to access specialised smoking cessation support. In 2011, the Ministry of Health established the Smoking Cessation Treatment Support Program to provide smoking cessation medications to people who smoke at no cost who apply to outpatient Smoking Cessation Clinics [15]. Currently, there are 362 registered smoking cessation clinics in Türkiye, 24 of these are located in Istanbul [16].

There has been limited investigation in Türkiye regarding the use of e-cigarettes as smoking cessation aids [17–24]. Only one quantitative study in Istanbul examined FPs views on e-cigarettes as a harm reduction strategy in 2022 [24]. Tanriover et al. (2022) found that majority (93.4%) of FPs do not recommend e-cigarettes to their patients for smoking cessation, whilst some agreed e-cigarettes to be healthier than regular cigarettes (22.5%), e-cigarettes can decrease levels of the risk of cancer (17.7%) and e-cigarettes can help people who smoke quit smoking [24]. Moreover, a study conducted by Arslan et al. (2020), evaluated the opinions of FPs in Türkiye on some tobacco products including e-cigarettes and their use [17]. Their study found that approximately 85% of FPs knew what an e-cigarette was, thought them to be addictive and believed their use is prohibited in closed public areas however, only 18.9% knew that e-cigarettes contain nicotine and other chemicals [17].

This qualitative study follows a survey of FPs knowledge, attitudes, and perceptions of e-cigarettes for smoking cessation conducted by the authors. In the qualitative study we aimed to explore FPs knowledge, attitudes, beliefs, confidence levels and factors that influence recommendations to patients about e-cigarettes, particularly their use in the management of smoking cessation.

## Methods

### Study design

The consolidated criteria for reporting qualitative research (COREQ) guidelines were used to report the findings and describe the study methods [25].

This qualitative study involved in-depth, semi-structured interviews with Turkish FPs in Istanbul to understand perceptions of e-cigarettes and their use as smoking cessation aids [26]. Qualitative research has the potential to provide deeper levels of information than quantitative data alone and can provide context and explanation to findings arising from quantitative research [26].

### Research team and reflexivity

This research is part of a doctoral study of the lead author (MS) who has a background in Health Sciences and Health Policy. MS has completed training on qualitative interviewing and is supervised by academics with qualitative research, respiratory epidemiology and public health speciality (CB, AEO, BE and SK). MS was hosted and supervised by AEO and SK at the Department of Public Health, Faculty of Medicine, Istanbul University to conduct interviews with Turkish FPs and extend her knowledge of primary care in Istanbul. MS is a non-smoker, non- vaper and has no affiliations with the tobacco industry, e-cigarette industry or pharmaceutical companies. MS is fluent in Turkish and was able to conduct and transcribe the interviews in Turkish.

Our approach to this research sits within the pragmatist tradition [27] and we do not take a position one way or the other whether FPs should or should not use e-cigarettes as part of clinical work to support smoking cessation. Throughout this research a reflexive research journal was kept to aid researcher reflexivity.

### Sampling and recruitment

Participants were selected from a previous survey study of *N* = 243 Turkish FPs in Istanbul which explored their knowledge, attitudes, beliefs and recommendations on e-cigarettes as smoking cessation aids [28]. A questionnaire was created from previous published literature and included 17 questions that assessed FPs knowledge about e-cigarettes, their safety and efficacy, their level of confidence and their recommendation of e-cigarettes as alternative smoking cessation aids [28]. FPs that had completed the survey were asked to register their interest in a further qualitative study at the end of the survey. Participants that were interested provided their email address for the lead author to contact them. As the survey was anonymous, survey responses were not linked or identified when providing contact details. FPs in Istanbul were also purposefully sampled and individually invited through other social media communication sources (WhatsApp™) with the help of co-authors (AEO and MTU). Participation was voluntary and all participants provided verbal consent to take part in the interviews at the time of the interview.

Twenty-four FPs were invited for an interview from those that expressed an interest to be interviewed. FPs that were purposefully sampled were asked to complete a questionnaire-based survey before being interviewed. In total six FPs and five FP trainees were interviewed (four from the survey and seven from other communication channels) between December 2022 and January 2023. Sampling ended when no new information was emerging impacting on the substantive interpretation of findings.

### Data collection

In depth semi-structured interviews were conducted one-on-one by the lead author in Turkish, and were guided by a semi-structured interview schedule. The approach of Minichiello et al. [29] was used to develop the interview schedule which was informed by the Theory of Planned Behaviour (TPB) [30], along with topics selected based on a review of the literature and preliminary findings found from the analysis of the Turkish survey study [7].

The interviews included discussions about smoking cessation in primary care settings (e.g. smoking cessation counselling, smoking cessation treatments and confidence levels about providing smoking cessation advice), beliefs and knowledge about e-cigarettes as smoking cessation aids and recommendations and confidence levels when having discussions with patients about e-cigarettes. The interview guide can be found in the supplementary file both in English and Turkish.

### Data analysis

Interviews were audio recorded and conducted online using Zoom™. They ranged in length between 13 and 50 min. The first three interviews were used to pilot test and refine the interview guide. Audio from the interviews was transcribed verbatim in Turkish by author MS and then translated to English following principles for qualitative interviewing in multiple languages [31]. Author BE, also bilingual in Turkish and English languages, checked the transcription and translation of audio from Turkish to English on the first three interviews. The authors met on several occasions during collection of data and then during the coding process to discuss the findings and evolving themes. Reflexive thematic analysis by Braun and Clarke [32] was used to analyse the data and QSR NVivo was used to aid coding and thematic analysis following the six steps outlined by Braun and Clarke. Key quotes that provide contextual information or exemplify ideas within themes were organised and retrieved from QSR NVivo for inclusion in the result section. Emerging themes were discussed with authors at regular review meetings. Both deductive and inductive approaches were used to analyse the data guided by the TPB. Authors agreed that after eleven interviews no further interviews were required as no new concepts were emerging.

## Results

### Description of the study sample

A total of eleven FPs and FP trainees were interviewed. The sample comprises of FPs located in six districts across Istanbul. An equal number of males and females were interviewed. The average age was 35 years and the average years of practice was six. The participants characteristics can be found in Table 1.

**Table 1:** Demographic characteristics

		Family physicians *n* = 6 (55%)	Family physician trainee *n* = 5 (45%)	Total *N* = 11
Gender	Female	*n* = 4 (36.3%)	*n* = 2 (18.2%)	*n* = 6 (54.5%)
	Male	*n* = 2 (18.2%)	*n* = 3 (27.3%)	*n* = 5 (45.5%)
Age (min 26 years-max 62 years)	20–30	*n* = 2 (18.2%)	*n* = 4 (36.3%)	*n* = 6 (54.5%)
	31–40	*n* = 2 (18.2%)	*n* = 1 (9.1%)	*n* = 3 (27.3%)
	60+	*n* = 2 (18.2%)		*n* = 2 (18.2%)
Years of practice (min 6 months-max 35 years)	0–10	*n* = 5 (45.5%)	*n* = 5 (45.5%)	*n* = 10 (91%)
	35+	*n* = 1 (9.1%)		*n* = 1 (9.1%)
Worked in a smoking cessation clinic	Current	*n* = 2 (18.2%)	*n* = 1 (9.1%)	*n* = 3 (27.3%)
	Previous	*n* = 1 (9.1%)		*n* = 1 (9.1%)
	No	*n* = 3 (27.3%)	*n* = 4 (36.3%)	*n* = 7 (63.6%)
Districts	*Eyüpsultan*		*n* = 1 (9.1%)	*n* = 1 (9.1%)
	Sancaktepe	*n* = 1 (9.1%)		*n* = 1 (9.1%)
	*Şişli*	*n* = 3 (27.3%)	*n* = 3 (27.3%)	*n* = 6 (54.5%)
	Sultanbeyli	*n* = 1 (9.1%)		*n* = 1 (9.1%)
	Sultangazi		*n* = 1 (9.1%)	*n* = 1 (9.1%)
	*Üsküdar*	*n* = 1 (9.1%)		*n* = 1 (9.1%)

All FPs stated they had people who smoke in their patient group and half (*n* = 5) reported patients using e-cigarettes. Four main themes were identified and described below.

#### Theme 1. insufficient knowledge and requests for support services about e-cigarettes as smoking cessation aids

All FPs expressed they did not have enough knowledge or up to date information about e-cigarettes to advise their patients on their use in quitting smoking.

*“To be honest*,* we do not have much knowledge on this subject*,* as FPs in general practice. I do not think we question this enough to patients in smoking cessation clinics.”* (FP trainee #9, F, Age 26).

*“I do not think I have enough knowledge about e-cigarettes. We know more information about regular cigarettes*,* but I may not be able to completely answer patient questions about e-cigarettes.”* (FP #10, F, Age 28).

A couple of FPs mentioned they “don’t have experience in this area and don’t have much information on this topic as they are novel products”. (FP #1, F, Age 35; FP trainee #7, F, Age 27)

*“We [FPs] do not know much information about this topic because this is a new product. Maybe there is information and research out there that I do not know about.”* (FP trainee #7, F, Age 27).

Some FPs expressed they felt there was a lack of research on e-cigarettes as an alternative smoking cessation aid and as such, e-cigarettes did not yet have a place in clinical settings.

*“As there is no research done in this area and we do not know the chemicals in these products*,* there is a potential for risk.”* (FP #1, F, Age 35).

*“I have not done much research in this area because we do not come across these types of cases often.”* (FP #10, F, Age 28).

FPs were keen to learn more about e-cigarettes and have “a little more knowledge to be able to guide patients better” (FP #10, F, Age 28). They wanted to see further research in this area.

*“Clearer information about the harms of e-cigarettes to the body can be gathered in studies. In particular*,* when comparing if e-cigarettes are less harmful than cigarettes. If I could gather a little more information about it*,* I would feel a little safer. Since we cannot obtain clear information*,* we cannot give positive evidence-based information [to patients].”* (FP trainee #3, M, Age 26).

In order to find a solution for FPs needs to help them when giving advice to patients about e-cigarettes, FPs suggested a range of support services and training that would be beneficial to them.

*“There may be videos*,* brochures and booklets on this*,* these are the things that will strengthen our knowledge.”* (FP #1, F, Age 35).

*“There are smoking cessation courses that provide certificates. Within this course a subheading that includes e-cigarettes would be nice. This topic is not covered in our education*,* I think it needs to have its own place.”* (FP trainee #7, F, Age 27).

#### Theme 2. FPs beliefs on the harms and concerns of e-cigarettes as smoking cessation aids

This sample of FPs held wide ranging views on the potential harm from e-cigarette use. Some thought e-cigarettes were “less harmful” (FP trainee #7, F, Age 27) and that e-cigarettes “can be used if successful” in helping people who smoke quit (FP #4, F, Age 32).

Other FPs thought that e-cigarettes were just as harmful as regular cigarettes, as it acts “like an imitation of cigarettes” (FP #4, F, Age 32) and continues the hand to mouth action (FP #8, M, Age 28; FP #4, F, Age 32). Some thought e-cigarettes continued the nicotine addiction, “addiction is addiction. I do not treat addiction by substituting it with something else” (FP #6, M, Age 60) and it “should be defined as harmful” (FP #1, F, Age 35).

FPs mentioned that e-cigarettes were not reliable products and “smoking should be ceased through using other methods” (FP trainee #9, F, Age 26) for people who smoke that want to quit. “As doctors, we can guide our patients more accurately using other methods” (FP trainee #9, F, Age 26).

*“Instead of e-cigarettes*,* other options in smoking cessation clinics [should be recommended]*,* such as therapy or psychotherapy*,* maybe even hypnosis. I think all of these are better than e-cigarettes. I think e-cigarettes are not right.”* (FP #4, F, Age 32).

*“I do not think it is reliable because I think this prohibited item is produced behind closed doors. The amount of nicotine in it is not at the required dose*,* everyone uses different flavours*,* there are different doses of nicotine. I do not believe in its reliability.”* (FP #6, M, Age 60).

FPs held concerns about e-cigarettes enabling “never smokers to start vaping” (FP trainee #2, M, Age 29) and having e-cigarettes appeal to younger adults for their various flavours, aromas and packaging. This was a concern to FPs as they believed “it encourages people in the younger age group” (FP trainee #3, M, Age 26) to start e-cigarettes and “increases the rate of smoking in society” (FP trainee #3, M, Age 26).

The potential for dual use and e-cigarettes “starting a gateway effect” (FP trainee #9, F, Age 26) and “a role as a step in the transition to smoking” (FP trainee #3, M, Age 26) and other tobacco products was also a concern for FPs.

*“I think it can trigger dual use. They both have nicotine in them. The patient would be ingesting more nicotine*,* the tolerance would increase. Patients shouldn’t be using both products at once.”* (FP trainee #7, F, Age 27).

*“The biggest concern I have is that once patients quit smoking they will switch to e-cigarettes*,* which makes it addictive. I think that e-cigarettes are used as an escape method.”* (FP #10, F, Age 28).

#### Theme 3. recommendations on e-cigarettes and policies for prescribing nicotine e-liquids

Almost all FPs in the study stated they would not recommend e-cigarettes to their patients as smoking cessation aids. Some were hesitant, “I am unsure on whether or not I would [recommend e-cigarettes], as I do not know enough about this” (FP #8, F, Age 28) and others stated if evidence shows e-cigarettes can help with quitting smoking this is when they would think about recommending them.

*“Maybe if there is research or evidence about this that shows e-cigarettes do help people to quit*,* then I would [recommend them].”* (FP trainee #3, M, Age 26).

*“If there is scientific evidence*,* I may recommend them*,* but I would remain sceptical. I cannot say I would recommend them completely.”* (FP #10, F, Age 28).

Half of FPs interviewed were not keen on a prescription model for e-cigarettes, where nicotine e-liquids could be prescribed as part of a smoking cessation plan (FP #4, F, Age 32; FP trainee #5, M, Age 32; FP #8, F, Age 28; FP trainee #9, F, Age 26; FP #10, F, Age 28; FP #11, M, Age 62). One thought, “A person who has the intention to quit should not try to quit with something similar to cigarettes.” (FP #8, F, Age 28) and another said, “I prefer removing the whole thing from a patient’s life rather than replacing it with something else” (FP #10, F, Age 28).

When asked ‘why’ FPs would not prescribe or recommend e-cigarettes as a treatment for smoking cessation, many said they would only consider prescribing e-cigarettes if strong evidence and research showed they helped people who smoke quit and if they were authorised by the government.

*“I will only consider it as a result of proper research that proves e-cigarettes are safer compared to cigarettes. I will consider prescribing if it has been proven and authorised for prescription.”* (FP trainee #2, M, Age 29).

*“If there is scientific data*,* then yes*,* no matter what*,* e-cigarettes are products that have very little harm compared to cigarettes*,* and those who use it can definitely quit.”* (FP #6, M, Age 60).

#### Theme 4: lack of confidence to comfortably answer patient questions about e-cigarettes

While this sample of FPs felt confident when giving advice about smoking cessation they did not have the confidence to answer patient questions about e-cigarettes if asked. This was mainly due to not having the requisite knowledge and information about e-cigarettes.

*“I do not feel very confident. Our assurance is evidence and scientific data. If this is supported by scientific studies*,* I would feel confident in this regard.”* (FP trainee #2, M, Age 29).

*“I do not feel very confident because I do not feel very well equipped about it. At the moment*,* I can make observation-based comments on people around me or other patients.”* (FP trainee #3, M, Age 26).

A couple of FPs expressed they would like to receive “more training and education in this area” (FP #1, F, Age 35) and “more knowledge on this subject and receive more training and guidance” (FP #9, F, Age 26) to increase their knowledge and give effective advice to patients about e-cigarettes as a form of smoking cessation.

Other FPs wanted more training available to them in smoking cessation to enable them to feel more confident.

*“We do not get much training at smoking cessation clinics*,* especially as FPs. I would like to attend a certified course/training program that provides a certificate upon completion on cigarettes and e-cigarettes in general practice. I think it would be better if it was made compulsory for us [FPs].”* (FP #10, F, Age 28).

## Discussion

FPs in Istanbul, a large and heavily populated city in Türkiye, had little to no clinical knowledge about vaping and this impacted their confidence to discuss e-cigarettes with their patients. They had no knowledge of current published research describing the role of e-cigarettes as a smoking cessation aid despite feeling confident about their understanding of smoking cessation more generally. The participants in this study were keen to learn more about e-cigarettes and receive formal training and education to be able to inform their patients and give advice about e-cigarettes, including in the context of their use in smoking cessation.

FPs in Istanbul held a variety of views on the harms of e-cigarettes compared to regular cigarettes [7]. Some believed they were less harmful whilst others thought they were just as harmful as combustible cigarettes because they contained nicotine and other chemicals. Furthermore, they held concerns that e-cigarettes could act as a gateway to cigarettes especially among younger age groups and other tobacco products by current non-smokers, as well as dual use. These concerns have been reported amongst FPs in other countries such as Australia which also bans the sale of e-cigarettes but has adopted a prescription model for access to nicotine containing e-liquids [8].

Our findings are in line with other studies globally and highlight the need for more evidence on the safety and efficacy of e-cigarettes as a smoking cessation aid [7, 33–38]. Turkish FPs, like physicians in other countries, felt there was a lack of reliable information about e-cigarettes and not enough evidence to be worth recommending them for use as smoking cessation aids [7]. Most FPs were not prepared to prescribe e-cigarettes to patients. In Türkiye, unlike some other countries, there is no allowance for the prescription of e-cigarettes and participants would only consider prescribing e-cigarettes if they are to be authorised and approved by the Turkish government.

A key barrier for most health professionals experience when it comes to discussing smoking cessation with patients is the lack of knowledge and skills in regards to tobacco and tobacco control [12, 17]. Our FPs were not confident in their abilities to discuss e-cigarettes with their patients or did not feel comfortable to do so due to insufficient evidence and lack of knowledge.

Information and resources from trusted, reliable sources are needed. Brochures, videos and pamphlets were desired among FPs to help them give advice about e-cigarettes to patients along with formal training leading to certificates or recognition of training in this area. Smoking cessation programs are available to FPs who are interested in educating themselves further in this area, after completing their specialty training as a family physician. Some of our FPs also mentioned the process should be made easier for them to be included in smoking cessation courses and should be made compulsory in their specialty training as FPs.

### Limitations

The results from this study offer valuable insights into the experiences of Turkish FPs and their perceptions of e-cigarettes as smoking cessation aids. As is typical of qualitative research, the findings reflect the experiences of a small sample of purposefully selected respondents and cannot be generalised to all FPs in Istanbul. Six participants were located from the same clinical practice which may have influenced the findings due to shared perspectives. Our sample of FPs average years of practice was six and five of our participants were FP trainees. Moreover, not all of our FPs worked or had experience working in a smoking cessation clinic, thereby contributing to lack of knowledge in understanding the use of e-cigarettes as smoking cessation aids. More experienced FPs and those with more experience working in smoking cessation clinics may have different experiences and perspectives to those expressed in our sample.

## Conclusion

To our knowledge this is the first qualitative study to have explored FPs knowledge, attitudes, and recommendations about e-cigarettes as smoking cessation aids in Türkiye. FPs had limited knowledge about e-cigarettes and were sceptical of the published literature on this topic. They were not willing to recommend e-cigarettes or prescribe nicotine e-liquids unless strong evidence and research showed them to be effective to quit smoking and were authorised by the Turkish government. FPs desired more information and resources from trusted sources to support them to learn about e-cigarettes in order to discuss them with their patients. Future research should consider collecting data from other health professionals and those that work in smoking cessation clinics as they have greater experience in this area and are exposed to patients who smoke. Further research should also sample participants from other cities and regions of Türkiye which may have different social and demographic populations to fully understand the experiences and needs of FPs in these regions in supporting smoking cessation amongst their patients.

## Electronic supplementary material

Below is the link to the electronic supplementary material.

Supplementary Material 1

Supplementary Material 2

## Data Availability

No datasets were generated or analysed during the current study.

## Abbreviations

COREQ: Criteria for reporting qualitative research
e-cigarettes: Electronic cigarettes
FCTC: Framework convention on tobacco control
FPs: Family physicians
TPB: Theory of planned behaviour
WHO: World health organization
