# Supplementary material for: Perceptions of family physicians in Istanbul about e-cigarettes as smoking cessation aids: a qualitative study
Source: Addict Sci Clin Pract. 2024 Dec 30;19:99. doi: 10.1186/s13722-024-00532-z (PMC11684242; doi:10.1186/s13722-024-00532-z)
Supplement: Supplementary file 1 — Supplementary Material 1 [file 13722_2024_532_MOESM1_ESM.pdf]

Interview Guide

Perceptions of family physicians in Türkiye about e-cigarettes as smoking cessation aids

<u>Introduction and general information</u>

Theme 1. Firstly, can you tell me a little about your clinical practice and the patients you see?

Probe: How long have you worked at this clinic?

Probe: Do you have any fields of special interest?

<u>Current practice on smoking cessation</u>

Theme 2. Of your patient group, do you have many who are smokers? Tell me a bit about them.

Probe: How do you identify them?

Is smoking status routinely recorded in practice software?

Do you provide smoking cessation counselling at your family practice clinic? If not, can you explain why?

Probe: Are GPs best to provide smoking cessation counselling, or do you refer to other organisations

Probe: What types of smoking cessation methods do you recommend to help your patients quit smoking?

Probe: How confident do you feel providing smoking cessation advice?

Probe: What would be needed for you to feel more confident?

<u>Patient discussions</u>

Theme 3. Of your patient group, do you have many who use e-cigarettes or are vaping? Tell me a bit about them.

Probe: What types of discussions have you had with patients about e-cigarettes?

Probe: Can you give examples of what you would say if you were asked about e-cigarettes?

Probe: Are GPs best to provide counselling about e-cigarettes or do you refer to other organisations?

Can you give me an example of when a patient asked you about vaping, or perhaps, when you spoke with a patient about their vaping?

<u>Beliefs about e-cigarettes</u>

Theme 4. What are your overall thoughts about e-cigarettes as a smoking cessation aid?

Would you recommend e-cigarettes to your patients?

Probe: If yes, why?

Probe: If no, why not?

What do you think about the relative harms, risks and safety of e-cigarettes compared to smoking regular cigarettes?

Probe: What are your reasons for thinking this or why do you think this?

What types of concerns do you have about the use of e-cigarettes as a cessation aid?

What are your opinions about e-cigarettes potentially triggering dual use?

What are your thoughts about e-cigarettes causing a gateway effect to smoking and other tobacco products?

Knowledge about e-cigarettes

Theme 5. Do you feel you have enough knowledge on e-cigarettes to confidently answer patients' questions?

If Yes, where do you receive e-cigarette information from and what types of evidence do you rely on?

If No, what information and guidance would you like to see on e-cigarettes to help you in your current practice?

Prescribing and policy on e-cigarettes

Theme 6. If Türkiye implemented a prescription policy that allowed doctors to prescribe e-cigarettes to their patients for smoking cessation, would you prescribe them?

Probe: If yes, why?

Probe: If no, why not?

Confidence and comfort about e-cigarette advice

Theme 7. How supported/comfortable/confident do you feel in giving advice about e-cigarettes to your patients?

Probe: Do you feel you know enough to comfortably prescribe e-cigarettes to your patients or do you feel as if you require further information and training in this area?

What other support services, programs or information would you like to see from your government or other non-government organisations?

Finally, do you have any other issues or concerns around e-cigarettes as smoking cessation aids that you wish to raise?
