# Supplementary material for: Perceptions of family physicians in Istanbul about e-cigarettes as smoking cessation aids: a qualitative study
Source: Addict Sci Clin Pract. 2024 Dec 30;19:99. doi: 10.1186/s13722-024-00532-z (PMC11684242; doi:10.1186/s13722-024-00532-z)
Supplement: Supplementary file 2 — Supplementary Material 2 [file 13722_2024_532_MOESM2_ESM.pdf]

Nitel görüşme kılavuzu

Türkiye'deki aile hekimlerinin e-sigara aracılığı ile sigarayı bırakma algıları

Giriş ve genel bilgiler

Tema 1. Öncelikle klinik uygulamanızdan ve gördüğünüz hastalardan biraz bahseder misiniz?

Probe: Ne kadar süredir bu birimde çalışıyorsunuz?

Probe: Özel ilgi alanlarınız var mı?

Sigarayı bırakma konusunda güncel uygulama

Tema 2. Hasta grubunuzdan çok sigara içen var mı? Bana biraz onlardan bahseder misiniz?

Probe: Onları nasıl tanımlarsınız?

Sigara içme durumu, uygulama yazılımında rutin olarak kaydediliyor mu?

Kendi aile sağlığı birimizde sigara bırakma danışmanlığı veriyor musunuz? Vermiyorsanız nedenini açıklar mısınız?

Probe: Sizce aile hekimliği birimleri sigara bırakma danışmanlığı sağlamak için en uygun yerler midir, yoksa başka kuruluşları mı tercih edersiniz?

Probe: Hastalarınızın sigarayı bırakmalarına yardımcı olmak için ne tür sigara bırakma yöntemlerini önerirsiniz?

Probe: Sigara bırakma tavsiyesi verirken kendinizi ne kadar emin hissediyorsunuz?

Probe: Kendinizi daha emin hissetmeniz için ne gerekir?

Hasta tartışmalar

Tema 3. Hasta grubunuzdan e-sigara kullanan var mı? Bana biraz onlardan bahseder misiniz?

Probe: Hastalarinizla e-sigarayla ilgli ne tür tartışmalar yaptınız?

Probe: Hastanız size e-sigara konusunda soru sorursa, ne diyeceğinizi örnek verir misiniz?

Probe: Aile hekimleri e-sigaralar hakkında danışmanlık sağlamak için en iyisi mi yoksa başka kuruluşları mı tercih edersiniz?

E-sigara hakkındaki inanışlar

Tema 4. Sigarayı bırakma yardımı olarak e-sigaralar hakkında genel düşünceleriniz nelerdir?

E-sigarayı önerirmisiniz?

Evet ise, neden?

Hayir ise neden?

E-sigaraların zararları, riskleri ve güvenliği hakkında ne düşünüyorsunuz?

E-sigaraların bırakma yardımı olarak kullanılmasıyla ilgili ne tür endişeleriniz var?

E-sigaralarin ve sigaranin birlikte kullanim riski olabilirmi? Bu konuda ne dusunyorsunuz?

E-sigara, sigara ve diğer tütün ürünlerine baslatabilirmi? Tetikleyicisi olabilirmi?

E-sigara konusunda bilgi

Tema 5. Hastaların sorularını güvenle yanıtlamak için e-sigaralar konusunda yeterli bilgiye sahip olduğunuzu düşünüyor musunuz?

Evet ise, e-sigara bilgilerini nereden alıyorsunuz ve ne tür kanıtlara güveniyorsunuz?

Hayır ise, biriminizde size yardımcı olması için e-sigara konusunda başka hangi bilgileri ve rehberliği görmek istersiniz?

E-sigara reçetesi ve politikası

Tema 6. Türkiye'de sigarayı bırakmak için hastalara e-sigara reçete yazma yetkisi olsaydi, yazar mıydınız?

Evet ise neden?

Hayir ise neden?

Tema 7. E-sigara tavsiyesi konusunda güven ve rahatlık

Hastalarınıza e-sigara konusunda tavsiye verirken kendinizi ne kadar desteklenmiş, rahat ve güvenli hissediyorsunuz?

Probe: Hastalarınızla e-sigara konusunu rahatça tartışacak kadar bilgili olduğunuzu düşünüyor musunuz veya bu alanda daha fazla bilgi ve eğitime ihtiyacınız var gibi mi hissediyorsunuz?

Başka hangi destek hizmetlerini, programları veya bilgileri hükümetinizden yada diğer sivil toplum kuruluşlarından görmek istersiniz?

Son olarak, e-sigaralarla ilgili dile getirmek istediğiniz başka sorunlarınız veya endişeleriniz var mıdır?
